# Tubulointerstitial nephritis and uveitis syndrome following meningitis and systemic lymphadenopathy with persistent *Toxoplasma* immunoglobulin M: a case report

**DOI:** 10.1186/s13256-021-02909-z

**Published:** 2021-09-23

**Authors:** Yoshihiro Oya, Hidekazu Futami, Takuya Nakazawa, Kazuyuki Ishijima, Keiko Umemiya, Fumiyoshi Takizawa, Naoki Imai, Hiroshi Kitamura, Ryutaro Matsumura

**Affiliations:** 1 Department of Rheumatology, Allergy and Clinical Immunology, National Hospital Organization Chibahigashi National Hospital, 673 Nitona-cho, Chuou-ku, Chiba City, Chiba 260-8712 Japan; 2 Laboratory of Autoimmune diseases, Department of Clinical Research, National Hospital Organization Chibahigashi National Hospital, Chiba City, Chiba 260-8712 Japan; 3 Department of Ophthalmology, National Hospital Organization Chibahigashi National Hospital, Chiba City, Chiba 260-8712 Japan; 4 Department of Pathology, National Hospital Organization Chibahigashi National Hospital, Chiba City, Chiba 260-8712 Japan; 5 Department of Internal Medicine, Seikeikai Chiba Medical Center, Chiba City, Chiba 260-0842 Japan

**Keywords:** Persistent immunoglobulin M (IgM), Drug-induced lymphocyte stimulation test (DLST), Lymphocyte transformation test (LTT), Tubulointerstitial nephritis and uveitis (TINU) syndrome, *Toxoplasma gondii*, IgG avidity test, Strain-specific diagnosis, Meningitis

## Abstract

**Background:**

Tubulointerstitial nephritis and uveitis syndrome is a rare lymphocyte-related oculorenal inflammatory disease presumed to be associated with drug use and infectious agents. *Toxoplasma gondii* is one of such pathogens that could exhibit encephalitis, meningitis, and uveitis in immunocompromised or in some immunocompetent individuals. If the immunoglobulin M of *Toxoplasma* is positive on screening, the interpretation of the result is not simple, especially when immunoglobulin M stays positive persistently.

**Case presentation:**

A 34-year-old Asian male developed fever, headache, and lymphadenopathy with tenderness, which was initially diagnosed as meningitis. Antibiotics were started, and diclofenac sodium was used for the fever. Although his symptoms were alleviated in a week by the treatment, gradual decline in renal function was noted, prompting a renal biopsy that indicated acute granulomatous interstitial nephritis. A week later, tenderness in both eyes with blurred vision appeared and revealed iritis and keratic precipitations in both eyes; hence, the diagnosis of acute tubulointerstitial nephritis and bilateral uveitis syndrome was made. *Toxoplasma gondii*-specific immunoglobulin G and immunoglobulin M titers were both positive. Although we could not rule out recent infection of *Toxoplasma gondii*, which may cause uveitis initially, *Toxoplasma* immunoglobulin G avidity test indicated a distant infection, which allowed us to rule out meningitis and uveitis as responsible for the complication of recent *Toxoplasma gondii* infection. Drug-induced lymphocyte stimulation test, or lymphocyte transformation test of diclofenac sodium, was solely positive among the tested drugs. Uveitis was alleviated only with ophthalmic steroid, and renal function returned to normal without administration of systemic steroid.

**Conclusions:**

We experienced a case of diclofenac-induced tubulointerstitial nephritis and uveitis syndrome. In ruling out infections, *Toxoplasma* immunoglobulin M was persistently positive, and *Toxoplasma* immunoglobulin G avidity test indicated a “distant” infection. From these two results, we ruled out recent infection. However, it should be noted that “distant” infection indicated by commercial immunoglobulin G avidity is still a multiplex profile consisting of reinfection, reactivation, and latent infection. Narrowing down the infection profile of *Toxoplasma* is challenging in some cases. Therefore, careful diagnosis and extended follow-up of such patients are needed.

## Background

Tubulointerstitial nephritis and uveitis (TINU) syndrome is a rare oculorenal multisystemic autoimmune disease that may occur in response to various environmental triggers, including drugs and microbial pathogens [1]. Since TINU syndrome was first described by Dobrin in 1975 [2], only about 200 cases have been recorded until 2018 [1]. Matsumoto has identified a total of 102 Japanese cases from literature in the period between 1977 and 2013 [3]. Patients with TINU syndrome can present with systemic symptoms (fever, fatigue, malaise, asthenia, or headache), uveitis (bilateral eye pain, redness, blurred vision, or photophobia), and tubulointerstitial nephritis (rise in serum creatinine and urinary protein). Noncaseating granulomata were found in 13% of renal biopsy specimen [4].

*Toxoplasma gondii* is an intracellular protozoan parasite with worldwide distribution that infects more than one-third of the global population [5]. The clinical presentation of this parasite varies from asymptomatic in healthy individuals to neurological symptoms (encephalitis [6], meningitis), ocular symptoms (uveitis, such as posterior uveitis) [6, 7], myocarditis, pneumonitis, or opportunistic infections mostly in immunocompromised hosts. Although the renal manifestation of *Toxoplasma* infection is not frequent [8–10], *Toxoplasma* infection is listed as a differential diagnosis of acute tubulointerstitial nephritis (TIN) [11] or of granulomatous interstitial nephritis [10]. Even in immunocompetent hosts, some (10–15%) [12–15] individuals develop acute systemic manifestations. Most frequent symptoms are lymph node enlargement (94.6%), asthenia (86.5%), headache (70.3%), and fever (67.6%). Retinochoroiditis (10.8%) is also present in the analysis of 37 immunocompetent adults with acute acquired toxoplasmosis [16]. The severity of subacute toxoplasmosis of the primary infection may vary depending on the virulence of the strain or the amount of inoculum [17]. Although most of the symptoms in immunocompetent adults are self-limited and last from a few weeks to months [16, 18], life-threatening pneumonia or death has also been reported [19].

The prevalence of recent infection of *Toxoplasma* in immunocompetent adults is best studied with pregnant women. Primary infection at early pregnancy is well known to cause congenital toxoplasmosis. The positive rate of both immunoglobulin G (IgG) and immunoglobulin M (IgM) specific to *Toxoplasma gondii* in pregnant women varies worldwide: New Zealand, South Korea, USA 0.1–0.5%, Japan 1.0–1.5% [20, 21], or 3.8% [22], Africa, and Eastern Mediterranean 2–5% [21]. To make matters more complicated, *Toxoplasma* IgM can persist for several months [23–27] or years [28] after the acute phase of primary infection (recent infection). *Toxoplasma* IgG avidity test is a key tool to distinguish “true” recent infection from distant infection, widely used for the screening of pregnant women. Among the initially screened IgM-positive pregnant women, as much as 59% (in Brazil) [29], 56% (in Turkey) [30], and 71.4% (in Japan) [31, 32] of them are reported to have a higher titer of IgG avidity, indicating distant infections of more than 5 months before [33]. This high frequency of persistent IgM in the initially screened IgM-positive population is one of the reasons for the lack of reliability in serological tests of *Toxoplasma* [34].

In the diagnosis of an autoimmune disease, differential diagnosis of dozens of systemic diseases including infections are required. Some infections are difficult to exclude because of symptoms similar to a suspected autoimmune disease. A combination of TINU syndrome and *Toxoplasma gondii* infection is one of them. If the IgM of *Toxoplasma*-specific antibody is present, the diagnostic processes become more challenging. Such cases might be misdiagnosed as TINU syndrome with coexisting active toxoplasmosis, or underdiagnosed as just toxoplasmosis. Differential diagnosis of TINU syndrome and interpretations of *Toxoplasma* serological tests in immunocompetent hosts will be discussed. The chances of encountering this combination might not be so rare considering the worldwide prevalence of *Toxoplasma gondii*.

## Case presentation

A 34-year-old Japanese male with a medical history of gastric ulcer for 20 years, regular use of esomeprazole for the last 3 years, no known allergies, and no family history of kidney or eye disease presented to his local emergency department with complaints of having 2 days of 40 ℃ fever, headache, myalgia, general fatigue, and vomiting. These symptoms developed 2 days after an incised finger wound with a small amount of bleeding due to an injury during his sewage plumber work. A rapid test for influenza A and B antigens was negative; owing to concern for bacterial infection, he was started on amoxicillin and ibuprofen. His symptoms did not improve for 5 days, and he was referred to the neurology department of another hospital on day 6 of illness. On admission, he was alert. The headache and neck pain were triggered by his movement. His temperature was 38.5 °C, heart rate 88 beats/minute, and blood pressure 133/69 mmHg. Although the finger exhibited no redness, swelling, or abscess formation, physical examination revealed bilateral cervical and inguinal lymphadenopathy with tenderness. As meningeal irritation signs, neck stiffness was positive, and Kernig sign was positive at 60 degrees. No other neurological deficits were noted. White blood cell (WBC) count was 8200/mm^3^, with 70% neutrophils, 12.5% lymphocytes, 6.0% eosinophils, and 9.3% monocytes. Serum creatinine (Cre) was 0.91 mg/dL (80 μmol/L), and blood urea was 6.0 mg/dL (2.1 mmol/L). Erythrocyte sedimentation rate was 55 mm/hour, and serum C-reactive protein (CRP) was 11.58 mg/dL. Urine was negative for glucose and protein, the sediment contained < 1 white cell and < 1 red cell per high-power field, and two blood cultures were negative. Computed tomography (CT) scan of the brain did not identify any source of fever. A lumbar puncture was also performed in the neurology department. His cerebrospinal fluid (CSF) was clear and colorless. Initial pressure was high at 235 mmH_2_O. CSF cell count was 1 × 10^6^ cells/L (1/μL) without red blood cells, glucose level was 3.66 mmol/L (66 mg/dL) [plasma glucose level 5.55 mmol/L (100 mg/dL)], and protein level was 0.31 g/L (31 mg/dL); no organisms were observed on Gram stain. No bacterial growth was detected in bottles of CSF and in bottles of blood. Then, treatment with oral levofloxacin 500 mg/day was initiated on day 6 of illness. Nonsteroidal antiinflammatory agents/drugs (NSAIDs), that is, diclofenac sodium suppositories 25 mg, were also administered three times a day for a week until remission of high fever on day 13 of illness (Fig. 2c). After the initiation of the treatment, contrary to the improvement of the inflammatory findings and parameters, kidney function deteriorated. Serum CRP levels were 11.58, 9.01, and 0.67 mg/dL, and serum Cre levels were 0.98, 1.28, and 2.74 mg/dL on day 6, 9, and 17 of illness, respectively. WBC count was 10,600/mm^3^, with mild elevated 12.5% eosinophil (reference value < 6.0%) on day 11. There was no skin eruption on extremities. Levofloxacin and esomeprazole were discontinued on day 17, and rapid reduction of kidney function prompted a transfer to our hospital on day 20 of illness for further evaluation and management (Fig. 2a,b). On admission, he had no fever and no complaints. There was no weight increase or pretibial pitting edema. The finger cut had healed without any scars or redness, and bilateral cervical and inguinal lymphadenopathy had diminished with little tenderness. Laboratory studies showed a WBC count of 10,000/mm^3^, with 60% neutrophil, 25% lymphocytes, 8.0% eosinophils, and 4.0% monocytes. Erythrocyte sedimentation rate was 22 mm/hour, serum CRP was 0.85 mg/dL, serum Cre was 2.09 mg/dL (185 umol/L), blood urea was 27.3 mg/dL (9.4 mmol/L), serum beta-2-microglobulin was 3.8 mg/L (reference value < 2.0 mg/L), urinary beta-2-microglobulin was 1589 ug/L (reference value < 229 ug/L), and urinary *N*-acetyl-β-d-glucosaminidase (NAG) was 7.9 IU/L (reference value < 6.9 IU/L). The results of laboratory tests showed that the levels of sodium, potassium, chloride, calcium, total protein, albumin, aspartate aminotransferase (AST), alanine aminotransferase (ALT), and uric acid were normal. IgG was 1073 mg/dL, immunoglobulin A (IgA) was 129 mg/dL, and IgM was 78 mg/dL. Urine remained negative for glucose and protein, and the sediment contained 0–1 white cell and 0–1 red cell per high-power field. Serum cystatin C measurement was 1.79 mg/L (reference value 0.57–1.01 mg/L), and glomerular filtration rate (GFR) was 61 mL/min/m^2^. His fractional excretion of sodium (FeNa) was elevated to 2.1% (reference value < 1%). Renal sonography revealed normal level in resistance index.

**Fig. 1:**
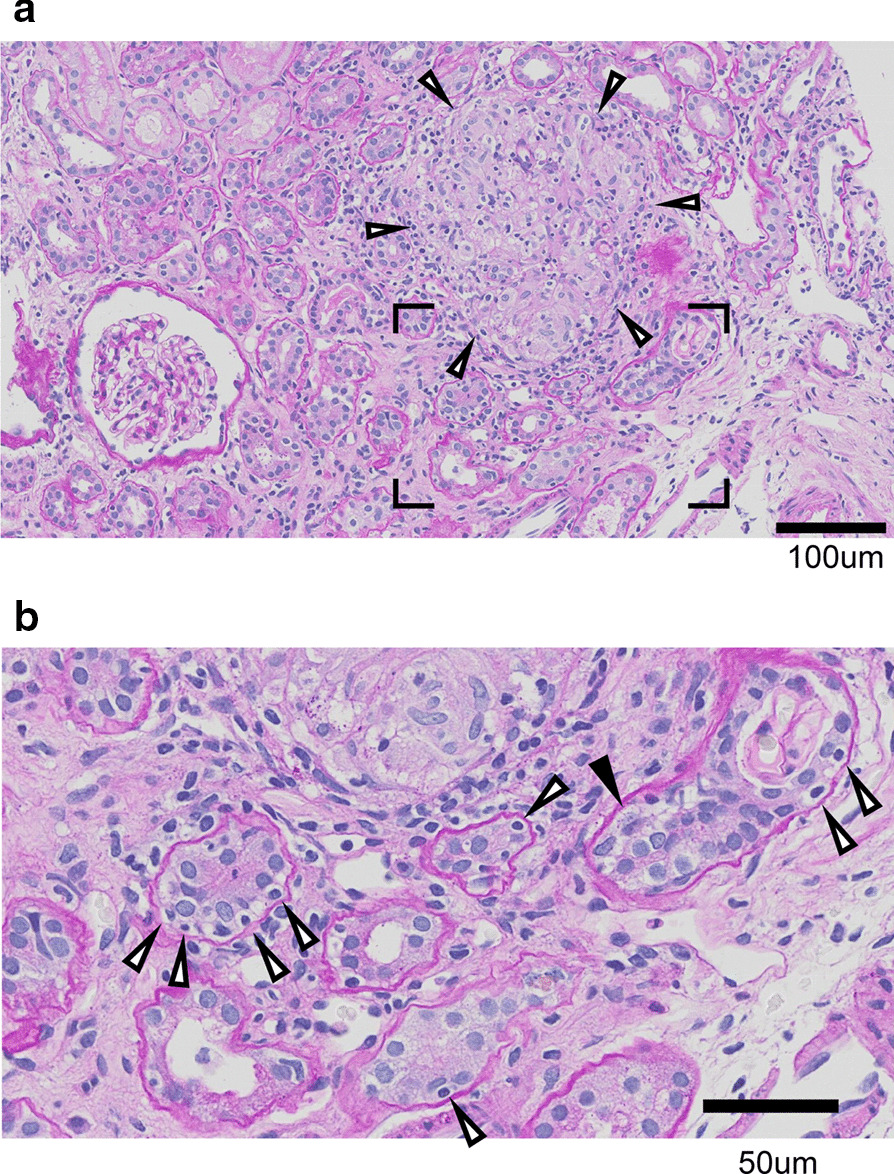
Renal biopsy. **a** Light microscopy of the renal biopsy (periodic acid–Schiff stain) revealed normal glomerulus with formation of interstitial granulomas indicated by arrow heads. Granulomas were composed of lymphocytes, plasma cells, macrophages, epithelioid cells, and multinucleated giant cells. Bar = 100 μm. **b** Magnified picture of marked area in **a**, revealing a focal distribution of mononuclear cell infiltrates in the interstitium, associated with focal tubular atrophy, with tubulointerstitial edema as indicated by black arrow head, and with mild tubulitis with intraepithelial lymphocytes, as indicated by white arrow heads. The moderate diffuse interstitial inflammation is composed of lymphocytes and eosinophils. Bar = 50 μm

A percutaneous renal biopsy performed on day 22 of illness revealed focal or belt-like distribution of numerous mononuclear cell infiltrates in the interstitium, associated with focal tubular atrophy, tubulointerstitial edema, and mild tubulitis. The moderate diffuse interstitial inflammation was composed of lymphocytes and eosinophils with mild fibrosis (Fig. 1b). Four interstitial granulomas composed of lymphocyte, plasma cell, macrophage, epithelioid cells, and multinucleated giant cells were identified (Fig. 1a). Glomerular and vascular structures were well preserved. Immunofluorescence showed no evidence of IgG, IgA, IgM, or immune complex deposition. Acid-fast staining for *Mycobacterium* was negative, and Grocott staining for detection of fungi was also negative. Angiotensin-converting enzyme (ACE) level was normal (11.4 U/L) (reference value 8.3–21.4 U/L). Chest X-ray and computed tomography did not reveal any abnormal masses, or mediastinum or hilar lymphadenopathy in the lung. Gallium scintigraphy did not show any abnormal accumulations. Saxon test results were normal (5.10 g/2 minutes) (reference value > 2.00 g/2 minutes). Anti-neutrophil cytoplasmic antibody (ANCA), MPO-ANCA, PR3-ANCA, anti-nuclear antibody (ANA), anti-SS-A/SS-B, anti-beta-2GPI, anti-RNP, anti-Sm, anti-dsDNA, anti-ssDNA, anti-Scl70, and anti-glomerular basement membrane (anti-GBM) antibodies were negative. IgG4 was at a normal level, soluble IL-2 receptor was 1050 U/mL (reference value 157–474 U/mL), and IgG-, IgA-, and IgM-specific antibodies against *Chlamydia* did not indicate a recent infection. Serologies for human immunodeficiency viruses (HIVs) 1 and 2, hepatitis B, and hepatitis C were negative. Anti-*Treponema pallidum* was negative, Epstein–Barr (EBV), mumps, and cytomegalovirus (CMV) serologies were not compatible with acute infection. According to these findings, we diagnosed the patient with granulomatous interstitial nephritis.

**Fig. 2.**
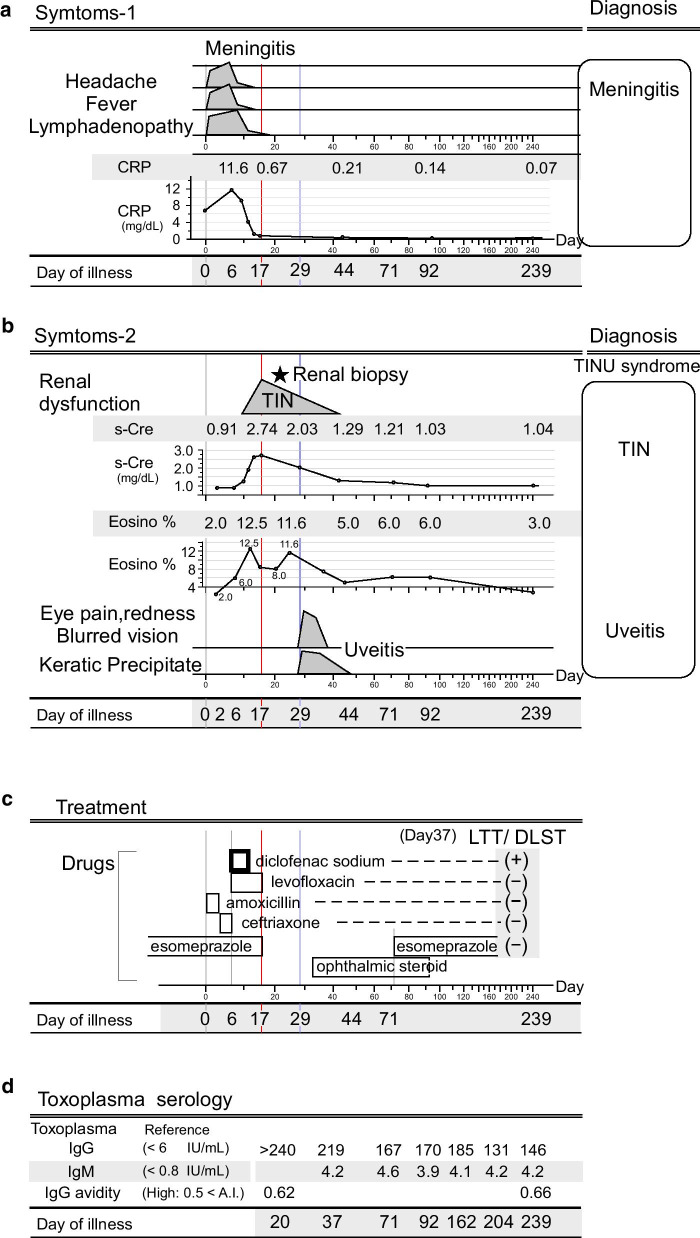
Summary of clinical course. **a**: Symptoms related to meningitis are shown. CRP levels are shown in the line chart. The *x*-axis shows day of illness (nonlinear scale). Onset of fever is defined as day 0 of illness. Key days are shown in the bottom line; day 6: initiation of diclofenac sodium; day 17: peak day of serum Cre (red line); day 29: development of uveitis (blue line); day 44: date of discharge; day 71: restart of esomeprazole; day 239: second sampling of IgG avidity test. The same time scale is shared by **a**, **b**, **c**, and **d**. **b**: Symptoms related to TIN and uveitis are shown. Serum Cre and percent peripheral eosinophils of the total WBC counts (eosinophils %) are shown in the line chart. ★: Renal biopsy was performed on day 22 of illness. **c** Duration of each administered drug is shown with rectangles. The patient had been administered esomeprazole for 3 years on day 0. The results of lymphocyte transformation test (LTT)/drug-induced lymphocyte stimulation test (DLST) (performed on day 37 of illness) are shown in parenthesis on the right side. The drug positive for LTT/ DLST is shown in bold line. **d** Values of *Toxoplasma gondii*-specific IgG, IgM, and IgG avidity along with the clinical course are shown in a table. The positions of the days of illness are adjusted to match the time scale of **a**, **b**, and **c**. High IgG avidity indicate a distant infection of more than 5 months before. IgG avidity test of day 20 (cryopreserved serum) and day 239 were performed together on day 239

On day 29 of illness, eye pain, conjunctival hyperemia, tenderness, photophobia, and blurred vision appeared in both eyes. Best-corrected visual acuity (BCVA) was 1.2 in the right eye and 1.5 in the left eye. Intraocular pressures were normal (right 11 mmHg/left 14 mmHg) with deep anterior chamber. Slit-lamp examination revealed iritis and keratic precipitation (corneal endothelial inflammatory precipitates/deposits) in both eyes. Anterior chamber cells were 1+ right and 0.5+ left. Dilated fundoscopic examination revealed that retina and vitreous body had nonspecific findings. No fever and no lymphadenopathy were observed. Hence, the diagnosis of acute tubulointerstitial nephritis and bilateral uveitis (TINU) syndrome was made, and ophthalmic steroid therapy was initiated (Fig. 2b, c). Symptoms of uveitis, eye pain, redness, and blurred vision disappeared in 1 week. Keratic precipitations were reduced in 1 week, and disappeared in 2 weeks. His serum Cre level gradually improved to 1.29 mg/dL on day 44 of illness without any systemic medical interventions, and systemic steroid therapy was not provided throughout the course of the disease. Mild elevation of eosinophils was also gradually improved. Percentage of peripheral eosinophils/total WBC count were 8.0%/10000, 11.6 %/6700, and 5.0%/7600 on day 20, 24, and 44 of illness, respectively.

To rule out toxoplasmosis, which could also develop into lymphadenopathy, meningitis, and uveitis, *Toxoplasma*-specific IgG and IgM titers were evaluated on day 37 of illness, and revealed to be both positive 219 IU/mL (reference value < 6 U/mL) and 4.2 IE/mL (reference value < 0.8 U/mL) respectively. He had a dog for a pet, but not a cat. Despite the positivity of *Toxoplasma* IgM antibody, the bilateral uveitis responded well to the ophthalmic steroid therapy, lymphadenopathy disappeared,and no signs of meningitis was observed. We decided that antimicrobial therapy was not needed, and he was discharged on day 44 of illness. Drug-induced lymphocyte stimulation test (DLST) or lymphocyte transformation test (LTT), performed by a commercially based clinical diagnostic testing service (SRL, Inc., Tokyo, Japan), confirmed that the patient had a negative stimulation index (SI) score for levofloxacin, esomeprazole, ceftriaxone, and amoxicillin, while he had a high SI score exclusively for diclofenac sodium of 207% (reference value < 180%).

After discharge, uveitis recurrence was not detected, and ophthalmic steroid was terminated after 2 months of use. However, the titer of IgM of *Toxoplasma* remained at a high level for an additional 6 months; we were not sure whether *Toxoplasma* infection was implicated in this TINU syndrome (Fig. 2d). To address the question, *Toxoplasma* IgG avidity test was performed with serum sample of day 20 of illness stocked in freezer, and with fresh serum sample from day 239 of illness. The test was performed by a laboratory company (SRL) using Platelia (TM) TOXO IgG AVIDITY (Bio-Rad) according to the manufacturer’s protocol. In brief, optical density (OD) indicating *Toxoplasma* IgG in serum was measured by enzyme-linked immunosorbent assay (ELISA) after dissociating the link between antibody and antigen. Urea was used as the dissociating agent. Target antigen of the ELISA was purified from *Toxoplasma gondii* RH strain. Avidity Index (AI) was measured by the ratio of OD(dissociating agent treated) to OD(dissociating agent untreated). IgG AI of the above samples was 0.62 (day 20) and 0.66 (day 239) (reference value: low AI < 0.4, mid 0.4 ≤ AI < 0.5, high 0.5 ≤ AI), indicating a chronologically distant infection of more than 5 months before fever onset. Hence, we determined that the pathogenesis of TINU syndrome in our case was not implicated with a recent infection of *Toxoplasma*.

No recurrence of renal dysfunction was observed in the 18 months of follow-up; serum Cre levels were 1.21, 1.03, and 1.04 mg/dL on day 71, 92, and 239 of illness, respectively. Urinary beta-2-microglobulin was reduced to 234 μg/L (reference value < 229 μg/L) on day 204 of illness. HLA typing showed HLA-A3101, HLA-A3303, HLA-B4002, HLA-B4403, HLA-DR0802, HLA-DR1302. HLA-DQB1 0302, and HLA-DQB1 0604, none of which was reported to have strong association with TINU syndrome.

## Discussion

To diagnose TINU syndrome, differential diagnosis of infections and dozens of other systemic diseases are required. Among infections, *Toxoplasma gondii* is a pathogen with considerable prevalence on a global scale. Since IgM of *Toxoplasma* is known to persist in many cases [32], the chances of encountering patients with TINU syndrome with *Toxoplasma* IgM like our patient might not be so rare. Our case illustrates the difficulties in determining the pathogenic contribution of *Toxoplasma gondii* in a case of lymphadenopathy and meningitis followed by TINU syndrome. IgG avidity test seemed to rule out recent infection of *Toxoplasma* and led us to conclude that TINU syndrome in our case was not implicated with a recent infection of *Toxoplasma*. However, this process of interpretation may not be enough for a precise understanding of the infection profile of *Toxoplasma* in similar situations. The limitations of current commercial serological tests are also discussed here.

### Diagnosis of TINU syndrome

A T-cell-mediated mechanism has been postulated for pathogenesis of TINU syndrome [1, 4, 35]. On analysis of kidney biopsy specimens from drug-induced tubulointerstitial nephritis (TIN), oligoclonal proliferation of drug-antigen-specific T cells was detected by staining of specific T-cell receptor beta-chain variable-region (TCR Vβ) [36]. The oligoclonal immune response is also reproduced *in vitro* with peripheral blood mononuclear cells from the same patients [36]. Also in TINU syndrome, under the same scenario, the proliferation of antigen-specific T cells initiates the adaptive immune response, which activates the humoral response with B cells. This process, delayed-type hypersensitivity reactions of type IV, causes immunopathologic damage to the uvea and renal interstitium [1]. Autoantibodies against modified CRP (mCRP) is proposed to be a marker for distinguishing TINU syndrome from drug-induced TIN [37, 38]. mCRP is presumed to be one of the common target autoantigens in renal and ocular tissues [37–39]. To demonstrate antigen-specific T cells, an *in vitro* assay called lymphocyte transformation test (LTT), or drug-induced lymphocyte stimulation test (DLST) is utilized as a useful diagnostic procedure for drug-induced TIN [40–43] and for TINU syndrome [44]. However, the sensitivity of this test for the detection of causative agents of drug hypersensitivity reactions is not adequate in some drugs (such as anti-tuberculosis drugs) [45]. Though the results of LTT/DLST may not be absolute [41, 42, 44, 46], we considered that our case was drug-induced TINU syndrome caused by diclofenac sodium. Diclofenac sodium was administered 5 days before the increment of serum Cre and 3 weeks before the development of uveitis, suggesting it is chronologically reasonable to infer that TIN and uveitis were both induced from diclofenac sodium. LTT/ DLST was performed as described by Pichler *et al*. [40]. In brief, 0.20 × 10^6^ whole peripheral lymphocytes were stimulated with titrated density of medicine in culture medium (complete RPMI1640 medium), together with separately prepared serum from the same patient. Cultures were started in triplicate. After 72 hours, [^3^H] thymidine was added and cultured for an additional 24 hours, and the thymidine uptakes were counted from the harvested cells.

The major histologic changes of tubulointerstitial nephritis (TIN) are interstitial edema and intraepithelial infiltration of inflammatory cells in renal tubules, accompanied with destruction of tubular basement membranes (TBM), called tubulitis [47]. The infiltrated cells consist of T lymphocytes, monocytes, eosinophils, and plasma cells [43].

In a review of 40 renal biopsies of patients with granulomatous formation (37 of them are TIN), sarcoidosis was present in 50% of the patients, and drug-induced TIN was present in 18% [48]. In an area where infection is a more likely etiology, microbial pathogens such as *Mycobacterium*, tuberculosis, fungi, bacteria, spirochetes, and parasites (*Leishmania*, *Toxoplasma*) are associated with granulomatous TIN [48]. ANCA-associated vasculitis, Crohn’s disease, and TINU syndrome can also cause granulomatous formation in renal interstitium [43, 48]. Histological findings of tubulitis are classified into three groups by immunohistochemistry [47]: (1) negative for antibodies and immune deposits [49, 50]; (2) positive staining of immune complexes along the TBM that sometimes associates with complement [51]; and (3) linear staining of the TBM, usually with IgG and complement. The third group is known as anti-tubular basement membrane (anti-TBM) disease [52, 53].

Detailed diagnostic criteria for TINU syndrome were developed by John T. H. Mandeville and James T. Rosenbaum [4]. These criteria adopt a probabilistic approach to the diagnosis using the presented clinical features [1]. Based on the criteria, our patient is diagnosed as “definite” TINU syndrome (Table 1). As is noted in the criteria, the diagnosis of TINU syndrome requires the presence of both acute TIN and uveitis, without other systemic diseases that can cause TIN and uveitis. The important differential diagnosis suggested by the combination of fever, lymphadenopathy, anterior bilateral uveitis, and granulomatous interstitial nephritis is as follows; sarcoidosis, Sjogren’s syndrome, systemic lupus erythematosus, granulomatous with polyangiitis (GPA), Behcet’s syndrome, Epstein–Barr virus–associated infectious mononucleosis, tuberculosis, bacterial/fungal infections, toxoplasmosis, and histoplasmosis [4, 54].

**Table 1:** Diagnostic criteria for tubulointerstitial nephritis and uveitis syndrome

The diagnosis of TINU syndrome requires the presence of both acute interstitial nephritis (AIN) and uveitis, without other known systemic diseases that can cause either interstitial nephritis or uveitis
Cases are categorized further as “definite,” “probable,” or “possible” on the basis of (1) the diagnostic criteria for AIN as defined below and (2) the clinical characteristics of uveitis as defined below
**Definite TINU syndrome**
• AIN diagnosed histopathologically or clinically (complete criteria) and typical uveitis
**Probable TINU syndrome**
• AIN diagnosed histopathologically and atypical uveitis
or
• AIN diagnosed clinically (incomplete criteria) and typical uveitis
**Possible TINU syndrome**
• AIN diagnosed clinically (incomplete criteria) and atypical uveitis
Diagnostic criteria for acute interstitial nephritis
• Histopathologic diagnosis: renal biopsy consistent with tubulointerstitial nephritis
• Clinical diagnosis:^a^ presence of the following criteria (a case is considered to have “complete criteria” if the three factors listed below are present; a case is considered to have “incomplete criteria” if fewer than three factors listed below are present):
1. Abnormal renal function (elevated serum creatinine or decreased creatinine clearance)
2. Abnormal urinalysis: increased beta-2 microglobulin, low-grade proteinuria [a level below that seen in patients with nephrotic syndrome (2+ or less on a semiquantitative test, or a spot urinary protein-to-urinary creatinine ratio of < 3, or < 3.0 g protein/24 hours in an adult or < 3.5 g protein/1.73 m^2^/24 hours in a child)], urinary eosinophils, pyuria or hematuria without infection, urinary white cell casts, or normoglycemic glucosuria
3. A systemic illness lasting > 2 weeks, characterized by a combination of the following symptoms and laboratory findings:
a. Signs and symptoms: fever, weight loss, anorexia, malaise, fatigue, rash, abdominal or flank pain, arthralgia, or myalgia
b. Laboratory findings: evidence of anemia, abnormal liver function, eosinophilia, or Westergren erythrocyte sedimentation rate > 40 mm/hour
Uveitis characteristics
• Typical
1. Bilateral anterior uveitis with or without intermediate uveitis or posterior uveitis
2. Onset of uveitis ≤ 2 months before or ≤ 12 months after AIN
• Atypical
1. Unilateral anterior uveitis or intermediate uveitis or posterior uveitis or a combination of these categories
2. Onset of uveitis > 2 months before or > 12 months after AIN

^a^If atypical clinical features are present, or if the renal disease does not improve after 6 weeks with appropriate therapy, a renal biopsy is recommended

For complete explanation of this criteria, please see full reference [4]. *AIN*, acute interstitial nephritis

Distinction of sarcoidosis from TINU syndrome is particularly difficult [1]. In 736 patients of sarcoidosis, the percentage of renal involvement (renal sarcoidosis) was only 0.7% [55]. Another retrospective analysis of 47 patients with renal sarcoidosis reported that 90% of them had intrathoracic lesions, 66% had proteinuria, and 55% had increased ACE [56]. The lymph node enlargement in sarcoidosis is typically firm, nontender, and freely movable [57]. As opposed to this clinical phenotype, our case had lymphadenopathy with tenderness and did not have hilar lymphadenopathy, proteinuria, elevated ACE, and other findings such as hepatomegaly, nephrocalcinosis, or nephrolithiasis on CT scan and on Gallium scintigraphy. Moreover, uveitis, lymphadenopathy, and TIN activity in our case were appreciably alleviated without using systemic steroid in the 18 months of follow-up, which is contrasting to the high relapse rate of renal sarcoidosis even after strong steroid therapy [56]. This clinical presentation made drug-induced TINU syndrome more likely than renal sarcoidosis. Long-term follow-up would be needed to confirm this presumptive diagnosis.

The first TINU syndrome cases were described as “Acute eosinophilic interstitial nephritis with uveitis” by Dobrin [2]. From this description, one can infer that eosinophils may play a certain role in the pathogenesis of TINU syndrome. Eosinophilia is included in the diagnostic criteria of TINU syndrome as one of the manifestations of acute interstitial nephritis [4]. Systemic eosinophilia is reported in 17% of TINU syndrome patients (21 of 122 cases) [4]. Mild elevations of eosinophils were observed also in our case. Interestingly, the level of peripheral eosinophils showed two peaks in the clinical course. These peaks were noticed when reviewing the data retrospectively. The first peak was recorded in parallel with the upregulation of serum Cre, or the development of TIN. The second peak was recorded around 4–5 days before the development of uveitis. These fluctuations of eosinophils might reflect the pathogenesis of TINU syndrome. Whether peripheral eosinophils can be used as a prognostic or activation marker of TINU syndrome symptoms should be investigated.

TINU syndrome is probably an underdiagnosed disorder [4]. TINU syndrome would not have been diagnosed if the association of interstitial nephritis and uveitis were not clinically evident, or if one of the symptoms had resolved by the time other symptoms developed. TINU syndrome cases are diagnosed in 0.2–2% of patients attending specialist uveitis services [58], and in 0.4% (*n* = 15/3830) of uveitis patients in Japan [59]. Drugs and infections have been proposed as the leading acquired risk factors for the development of TINU syndrome. Larger datasets from renal literatures also suggest that most cases of TINU syndrome are caused by drug-induced hypersensitivity reaction [58]. Contrary to the differential diagnosis described in the above paragraph, specific infective agents reported as being possibly associated with TINU syndrome include tuberculosis [60], EBV [61–63], herpes zoster reactivation [63], systemic toxoplasmosis [8], and generalized lymphadenopathy [8].

A review summarizing 133 TINU syndrome cases noted that the main drugs proposed as risk factors for TINU syndrome are nonsteroidal antiinflammatory agents (18%) and antibiotics (24%) [4]. There is a case of levofloxacin-related anterior uveitis, vitritis, and macular edema that required oral prednisone to return to baseline vision in an adult female [64]. The most common causative drug for uveitis is bisphosphonate, which is employed in the treatment of osteoporosis or of dystrophic disease involving the skeletal system. Bisphosphonates have been associated with uveitis, nonspecific conjunctivitis, episcleritis, and scleritis. Several antibiotics have been causally linked to uveitis, most notably rifabutin, sulfonamides, and fluoroquinolones. Sulfonamide-associated uveitis develops quickly, often as fast as 8 days [64]. Esomeprazole, a proton pump inhibitor (PPI), was resumed 2 months after discontinuation, without any complications thereafter. This indicates that esomeprazole had little effect on the pathogenesis of our TINU syndrome, although PPIs are common causative drugs for drug-induced TIN, like antibiotics and NSAIDs [65, 66]. Increased pH in stomach helps the survival of tachyzoites of *Toxoplasma gondii* [67]. In the view of susceptibility to ingested infectious agents, the high pH may make it difficult to completely deny the contribution of esomeprazole to pathogenesis.

TIN and uveitis symptoms are not always concurrent. The review summarizing 133 TINU syndrome cases noted that ocular symptoms were concurrent with systemic symptoms in only 15% of cases; in 21% of cases, uveitis occurred before systemic symptoms, occurring up to 2 months beforehand; in 65% of cases, uveitis occurred after systemic symptoms with a median of 3 months, up to 14 months [4, 58]. Based on the study, eye pain and redness were observed in 41% (*n* = 55/133), blurred vision in 10.5% (*n* = 14/133), anterior uveitis in 73% (*n* = 97/133), and keratic precipitation in 9.8% (*n* = 13/133) of the TINU syndrome cases [4]. Another review summarizing 2619 uveitis cases reported that viral infections were seen in 8.6% (*n* = 226/2619) and parasitic infections in 7.5% of cases (*n* = 198/2619), most of which were *Toxoplasma* infection (*n* = 173) [68]. Most viral infection-related uveitis anatomically localized in the anterior (81%, *n* = 183/226), whereas most parasitic infection-related uveitis localized in the posterior part (77%, *n* = 153/198). This suggests that the bilateral anterior uveitis observed in our case was more likely to be TINU syndrome or viral infection related rather than parasitic infection related.

Urinary beta-2-microglobulin is widely used as an interstitial damage marker. In our case, it was highly elevated in the acute phase and gradually reduced to a normal level in parallel with remission of the illness. Consistent with our case, a review reported that urinary beta-2-microglobulin levels were elevated in all cases of tested TINU syndrome (100%, *n* = 37/37) and persisted for months [4]. Persistent histological change was reported in the two tested cases even at second biopsy 9 months after the first diagnosis of TINU syndrome [69]. They suggest a requirement of immunosuppressive therapy in selected TINU syndrome patients [69]. Urine TNFα and IL-9 levels are proposed to be good biomarkers to improve the prebiopsy diagnosis for acute interstitial nephritis [70], because TINU syndrome is thought to be a lymphocyte-mediated immune response [1].

### Interpretation of *Toxoplasma* serology

The second most common cause of encephalitis deaths in the USA is *Toxoplasma gondii*, following herpesvirus [6]. A common cause of uveitis in immunocompetent hosts is *Toxoplasma gondii*. As many as 2% of *Toxoplasma*-infected patients exhibit ocular toxoplasmosis, which can be present either in the context of recently acquired infection (recent infection) or of reactivated disease as retinochoroiditis [71]. Retinochoroiditis scarring has been considered as a finding of congenital infection, but is increasingly recognized as a result of acquired recent infection [72]. Therefore, clinicians have a good chance of seeing an immunocompetent patient with subacute *Toxoplasma* infections. However, medical literature related to acquired toxoplasmosis in immunocompetent patients is not enough [16], probably because acquired toxoplasmosis has been considered to be basically self-limiting.

IgG avidity test of *Toxoplasma* was originally developed by Hedman and his associates in Finland [73]. Maturation of antibody affinity to target antigen occurs in the weeks or months after the primary infection. IgG avidity is initially low in the first 3 months (may remain to be low for 1 year), and achieves a higher level in the late phase of (distant) infection (4–5 months) [34]. IgG avidity is necessary as confirmatory testing to distinguish the recent infection of *Toxoplasma* from patients with persistent IgM or false positive IgM [32]. In our case, although positivity of IgG and IgM of *Toxoplasma* was obtained, we only periodically monitored the level of IgG and IgM. Despite the lack of headache, fever, and uveitis, the persisting IgM confused our interpretation of the serological results, and urged us to perform IgG avidity of *Toxoplasma*. The test is still in the research phase and not covered by health insurance in Japan. The IgG avidity test performed on the frozen sample of day 20 of illness indicated a distant infection. Therefore, complication of a recent infection of *Toxoplasma* on his pathogenesis was excluded accordingly.

**Fig. 3:**
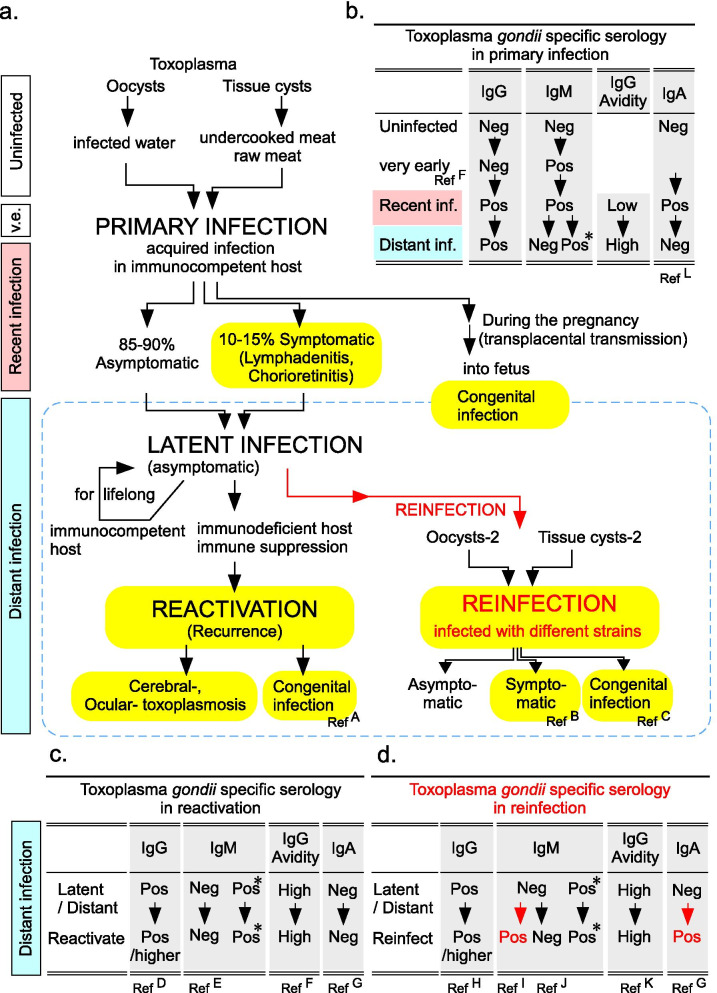
Toxoplasma serology tests in primary infection, reactivation, and in reinfection. **a** The transmission of *Toxoplasma gondii* to immunocompetent humans (intermediate host) occurs by ingestion of oocysts, normally via contaminated food or water. Infection can also occur via consumption of undercooked meat or raw meat containing tissue cysts with bradyzoites. The majority of cases (85–90%) are asymptomatic, but around 10–15% of the infected individuals develop systemic symptoms [12–15]. In either case, chronic (distant) infection can persist for the life of the hosts. If the hosts become immunodeficient or immune-suppressed, bradyzoites reactivate, which causes cerebral or ocular toxoplasmosis. If primary infection occurs during pregnancy, parasites can also infect the fetus by congenital transmission. Typical *Toxoplasma* serological changes of primary infection in immunocompetent patients are shown in **b**. The first isotype antibody to appear in the very early phase of the primary infection is IgM, followed by the appearance of IgG, which is required for confirmation of the infection. *Toxoplasma* IgG avidity test is a critical tool to distinguish recent infection from distant infection in pregnant women, because IgM of *Toxoplasma* is known to persist in many cases of distant infection [29–32] (persistent IgM is marked with * in this figure). However, congenital transmission also occurs in distant/latent infected host when the host becomes immunodeficient (**a**, lower left) or reinfected with different strains of *Toxoplasma gondii* (**a**: lower right). In such cases, commercial IgG avidities give high-level results even before the onset of reactivation or reinfection, because the hosts have been distantly/latently infected. Therefore, clinicians cannot interpret IgG avidity test as they do in primary infection. Clinically symptomatic phases are highlighted in yellow. Blue dot line indicates the “distant infection” profile, which consists of three infection profiles: latent infection, reactivation, and reinfection. Different strains of *Toxoplasma gondii* are described as oocysts-2 or tissue cysts-2. Representative changes of *Toxoplasma gondii* serology in reactivation and in reinfection are shown in **c** and **d**. In **d**, distinctive serological changes of reinfection from serum of reactivation are written in red letter. *IgG*, *IgM*, *IgA*, *Toxoplasma gondii*-specific IgG, IgM, IgA; *Inf*, infection; *Pos*, positive;* Neg*, negative; *Pos/higher*, positive or higher level; *v.e*, very early phase of infection. Recent infection is highlighted in pink, and distant infection is highlighted in light blue. This figure is modified from Vera Lucia Pereira-Chioccola *et al*. [15] and O Villard *et al*. [93]. *Ref* ^*A-K*^, reference group A–K; Ref. A: [90, 91], Ref. B: [96], Ref. C: [74–77, 78(p), 82(p), 94, 96]; p, presumed cases; Ref. D: [91], Ref. E: [91], Ref. F: [93], Ref. G: [74, 77, 82, 94], Ref. H: [74, 76, 77], Ref. I: [74, 91, 96], Ref. J: [74, 76, 77], Ref. K: [17, 93], Ref. L: [17]

A limitation of our investigation is that commercial *Toxoplasma* IgM and IgG tests cannot distinguish different strains of *Toxoplasma gondii*. Although reinfection of *Toxoplasma gondii* does not basically occur in immune-competent hosts except in some rare case reports [74–78], this may be true exclusively for the same strain. There are several strains in *Toxoplasma gondii* with different virulence, and prevalence varies depending on geographical factors [79, 80]. Contrary to the accepted notion above, a reinfection experiment in sheep model demonstrated that reinfection of a different strain of *Toxoplasma gondii* on preimmune sheep with the preceding infection does occur [81]. There is a case report of human reinfection by a different strain of *Toxoplasma* that was not protected by the immunity of the preceding strain of *Toxoplasma* [82] (Fig. 3a, lower right). The authors and others [83–86] also confirmed that acquired immunity against a preceding strain of *Toxoplasma* may not protect against reinfection by another strain, based on mouse model experiments. Although there is no evidence of increased risk of *Toxoplasma* infection for plumbers who are frequently exposed to sewage water [87], oocysts are persistent and prevalent in water, soil, and foods [88]. Also, vegetarians and certain occupations such as farmers are significantly more associated with *Toxoplasma* IgM seropositivity [89].

Besides reinfection, recurrence or reactivation of the primary infection is another type of “distant” *Toxoplasma* infection profile. Reactivation mostly occurs in immunosuppressed conditions [15]. Reactivation causes cerebral, ocular, or congenital toxoplasmosis [90, 91] (Fig. 3a, lower left). A retrospective serological analysis found that, among 217 cases of serologically positive ocular toxoplasmosis, only 3.2% were in recent primary infection profile, while 68.2% were in a distant (chronic) infection profile based on IgG avidity test [92]. Direct detection of *Toxoplasma gondii* is basically required for the confirmation of reactivated toxoplasmosis. For the detection, bioassay of suspected biological materials in laboratory mice or PCR tests targeting gene sequences of *Toxoplasma* are utilized [5]. Thus, the initial serological results of our case (positive IgG and IgM) indicate any of the following profiles: (1) recent primary infection; (2) latent/distant infection of primary infection (with persistent IgM); (3) recent infection of a different strain (reinfection); or (4) recurrence/reactivation of latent infection. The following serological results and the high IgG avidity in our case excluded the possibility of (1) recent primary infection. However, high titer and high avidity IgG generated by distant/preceding infections might mask the detection of low avidity IgG generated by a recent infection with a different strain. Therefore, the “distant” infection indicated by commercial IgG avidity test is still a multiplex profile consisting of (3) reinfection, (4) reactivation, and (2) latent infection [93]. Ranges of distant infection are described as light blue rectangle at left side in Fig. 3a.

The positivity of serum *Toxoplasma* IgA is reported to be a proof of reinfection and not of reactivation [74, 77, 82, 94]. In primary infection, *Toxoplasma* IgA appears shortly after IgM and persists for some time (usually 6–7 months) [95]. Reappearance of *Toxoplasma* IgM in previously infected patients is another serological marker of reinfection and not of reactivation [74, 91, 96], although this rule is applicable only in the absence of persistent IgM. However, reappearance of IgM is not always detected in reinfection-presumed cases [75–77]. Increased IgG of *Toxoplasma* observed at reinfection [74, 76, 77] or at reactivation [93] should be interpreted carefully, because such a rise is also observed in a delayed serological response to antigens after treatment of toxoplasmosis [97]. Serological variations in each infection profile are summarized in tables in Fig. 3b–d.

Evaluation of *Toxoplasma* IgA was not performed in our case. PCR detection from suspected biological materials was not attempted. The fact that the patient was not immunodeficient or immune-suppressed suggested that (4) reactivation was not likely. No remarkable change in *Toxoplasma* IgG throughout the clinical course suggested that (2) latent/distant infection was more likely than (3) reinfection or (4) reactivation. On the other hand, our case fortunately satisfied the diagnostic criteria for TINU syndrome. Based on the whole clinical course, we were able to interpret the serological results of *Toxoplasma* as (2) latent infection rather than (3) reinfection. It should be noted that making this diagnosis in the middle of the course was difficult. Protein microarray survey has identified a number of target antigens for IgG and IgM, from serum of *Toxoplasma*-infected patients [98]. Several efforts to identify strain-specific antigens [80, 83, 99–101] or stage-specific antigens [102–104] that enable more specific serotyping of *Toxoplasma gondii* have been reported. Future development of serological examination of *Toxoplasma* should facilitate the clinical diagnosis of autoimmune diseases such as TINU syndrome.

## Conclusion

This case illustrates the difficulty in determining the infection profile of *Toxoplasma gondii* in the diagnosis of TINU syndrome. Performing the IgG avidity test in a timely manner is recommended to determine the chronological assessment of the putative *Toxoplasma gondii* infection. However, these serological examinations still have limitations in their precise determination of the infection profile. Therefore, careful monitoring and extended follow-up of such patients are critical.

## Data Availability

Data sharing is not applicable to this article because no datasets were generated or analyzed during the current study.

## Abbreviations

TINU syndrome: Tubulointerstitial nephritis and uveitis syndrome
DLST: Drug-induced lymphocyte stimulation test
LTT: Lymphocyte transformation test
TBM: Tubular basement membranes
BCVA: Best-corrected visual acuity
KP: Keratic precipitation
NSAID: Nonsteroidal antiinflammatory agent/drug
CRP: C-reactive protein
Cre: Creatinine
CT: Computed tomography
CSF: Cerebrospinal fluid
WBC: White blood cell
NAG: *N*-acetyl-β-d-glucosaminidase
GFR: Glomerular filtration rate
FeNa: Fractional excretion of sodium
ACE: Angiotensin-converting enzyme
ANCA: Anti-neutrophil cytoplasmic antibody
ANA: Anti-nuclear antibody
GBM: Glomerular basement membrane
EBV: Epstein–Barr virus
CMV: Cytomegalovirus
SI: Stimulation index
HLA: Human leukocyte antigen
